# Bifrontal brain abscesses secondary to orbital cellulitis and sinusitis extension

**DOI:** 10.1186/s12245-016-0117-4

**Published:** 2016-07-26

**Authors:** David Traficante, Alexander Riss, Steven Hochman

**Affiliations:** Department of Emergency Medicine, St. Joseph’s Regional Medical Center, 703 Main St., Paterson, NJ 07030 USA

**Keywords:** Brain abscess, Emergency medicine, Infectious disease, Orbital cellulitis, Sinusitis

## Abstract

**Background:**

Intracranial abscesses are rare and life-threatening conditions that typically originate from direct extension from nearby structures, hematogenous dissemination or following penetrating cerebral trauma or neurosurgery.

**Findings:**

A 36-year-old male presented to our emergency department with complaints of left eye swelling, headache and drowsiness. On physical exam, the patient was febrile and his left upper eyelid was markedly swollen with fluctuance and drainage. Maxillofacial computed tomography was obtained to evaluate for orbital pathology but revealed bifrontal brain abscesses.

**Conclusions:**

Brain abscesses should be considered in the differential diagnosis for patients who present with the classic triad of headache, fever and neurological deficit.

## Findings

### Case synopsis

A 36-year-old Hispanic male presented to the emergency department (ED) with complaints of left eye swelling, headache and drowsiness. The patient had been seen two weeks prior to this visit at another emergency department for left eye swelling. At that time, he was diagnosed with a periorbital abscess and discharged home from the ED on a course of oral antibiotics. Over the following two weeks, the patient’s symptoms progressed to headache and increasing lethargy. The patient now also reported worsening left upper eyelid swelling with discharge and painful range of motion of the left globe. He reported no vision changes. In the ED, the patient was febrile, temperature was 102 °F. The left upper eyelid was swollen, erythematous, and fluctuant with pointing and purulent yellowish discharge. Visual acuity was 20/20 in both eyes. There were no focal motor or sensory deficits on exam. However, the patient did exhibit mental status changes including indifference to his current condition and a flat affect which was inconsistent with his baseline.

**Fig. 1:**
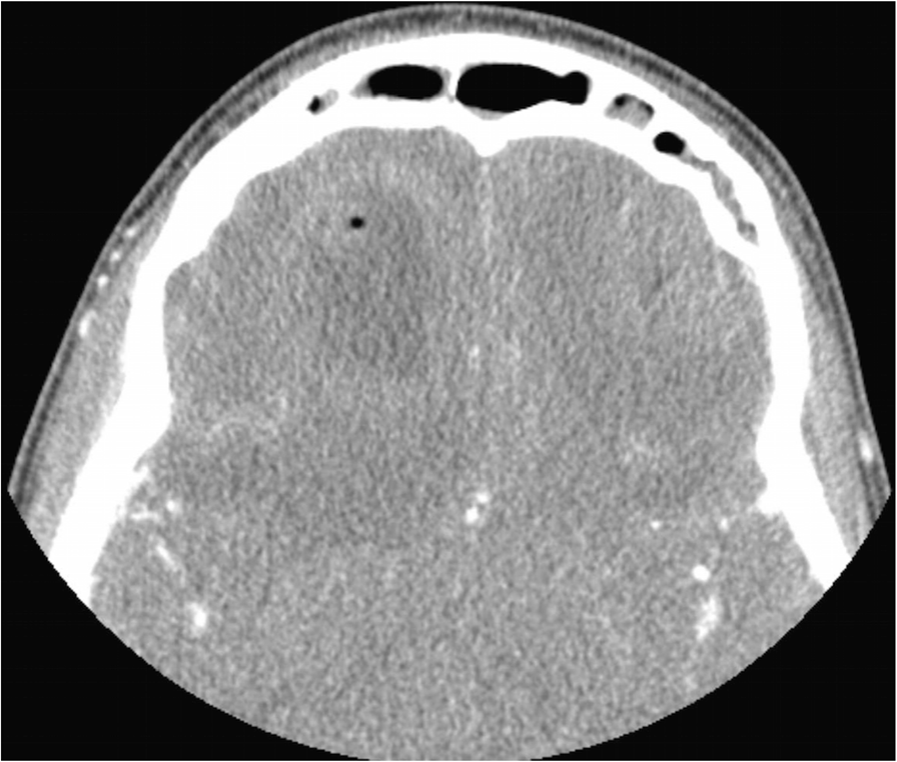
CT maxillofacial, axial view, demonstrating the finding of bifrontal rim-enhancing fluid collections

**Fig. 2:**
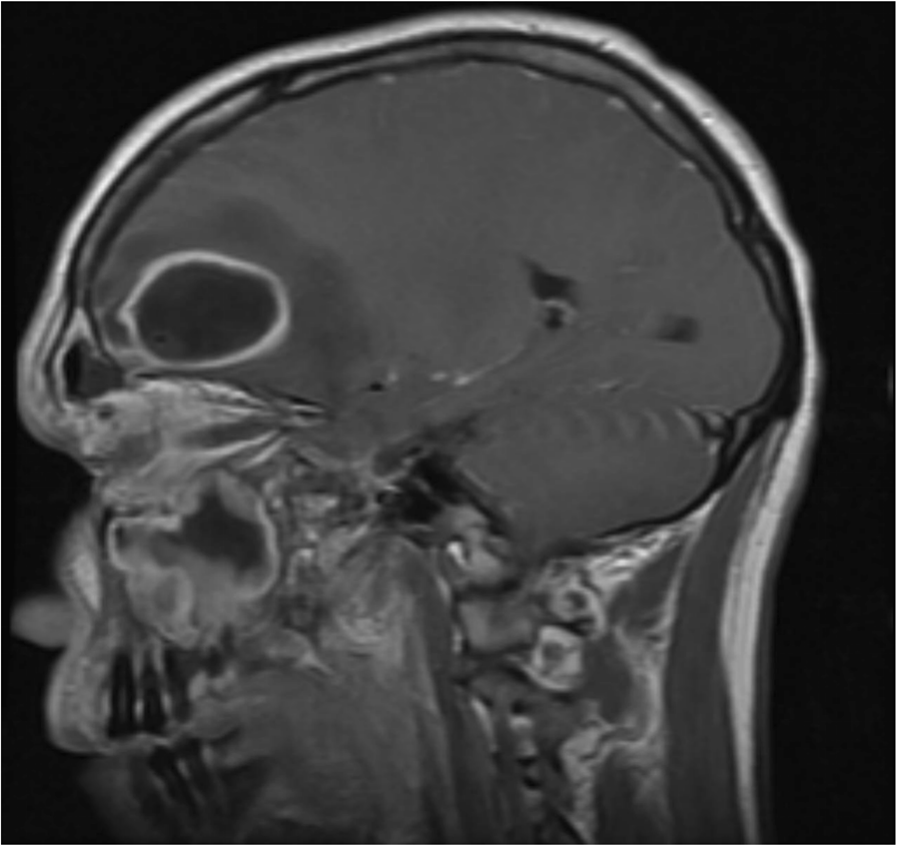
MRI brain, saggital view. Note the hyperintensity extending from the frontal sinus to the abscess cavity

**Fig. 3:**
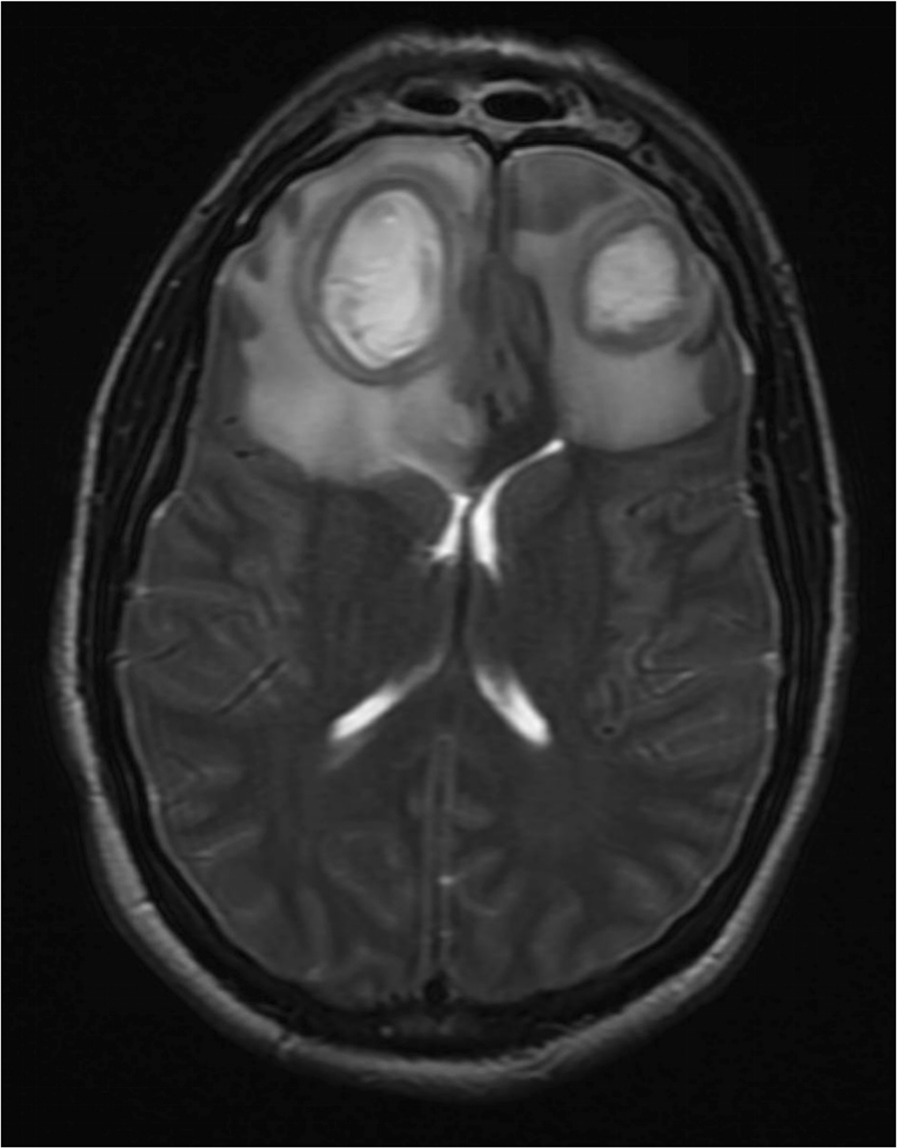
MRI brain, axial view

Computed tomography (CT) maxillofacial was initially obtained due to concern for orbital cellulitis and/or intraorbital abscess (Fig. 1). The study revealed two rim-enhancing fluid collections seen within the frontal lobes bilaterally. Magnetic resonance imaging (MRI) of the brain subsequently confirmed the presence of bifrontal brain abscesses, as well as left orbital cellulitis, periorbital abscess, and pansinusitis (Fig. 2, Fig. 3). The patient was managed with IV antibiotics, bilateral burr holes for brain abscess drainage followed immediately by bilateral endoscopic ethmoidectomy, antrostomy, sphenoid sinusotomy, frontal sinusotomy and incision and drainage (I&D) of the left upper eyelid abscess. Brain abscess cultures were positive for *Streptococcus anginosus* and *Prevotella intermedia*. The patient was seen in the ED six months after initial presentation for an unrelated complaint and at that time had no residual deficits.

### Bifrontal brain abscesses

Brain abscesses are focal pyogenic intracerebal infections which may present as life-threatening emergencies [1]. Infections can occur within the brain by direct extension from nearby structures, hematogenous dissemination or following penetrating cerebral trauma or neurosurgery [2]. Immunocompromised hosts are at particular risk, with etiologies in these patients commonly secondary to amebic or fungal infection. The classic triad for the clinical presentation of brain abscess includes headache, fever and focal neurological deficit, although the whole triad is seen in less than 50 % of cases [3]. This patient’s presentation with flat affect interestingly coincides with the psychopathology of the abscess location in the frontal lobes. Diagnosis is made by imaging studies including CT and MRI but is sometimes seen on radionuclide scans. Typically, images will reveal a ring-enhancing lesion with variable surrounding edema [4]. Treatment of brain abscesses requires a combination of drainage and antimicrobial therapy. Until gram stain results are available, antibiotic regimens should be based on the presumptive source of the infection.

## Abbreviations

ED, emergency department; I&D, Incision and drainage; CT, computed tomography; MRI, magnetic resonance imaging

## References

[1] Muzumdar D, Jhawar S, Goel A (2011). Brain abscess: an overview. Int J Surg.

[2] Bernardini GL (2004). Diagnosis and management of brain abscess and subdural empyema. Curr Neurol Neurosci Rep.

[3] Menon S, Bharadwaj R, Chowdhary A, Kaundinya DV, Palande DA (2008). Current epidemiology of intracranial abscesses: a prospective 5 year study. J Med Microbiol.

[4] Kastrup O, Wanke I, Maschke M (2005). Neuroimaging of infections. NeuroRx.

